# Analgesic antipyretic use among young children in the TEDDY study: no association with islet autoimmunity

**DOI:** 10.1186/s12887-017-0884-y

**Published:** 2017-05-16

**Authors:** Markus Lundgren, Leigh Johnson Steed, Roy Tamura, Berglind Jonsdottir, Patricia Gesualdo, Claire Crouch, Maija Sjöberg, Gertie Hansson, William A. Hagopian, Anette G. Ziegler, Marian J. Rewers, Åke Lernmark, Jorma Toppari, Jin-Xiong She, Beena Akolkar, Jeffrey P. Krischer, Michael J. Haller, Helena Elding Larsson, for the TEDDY Study Group, Marian Rewers, Kimberly Bautista, Judith Baxter, Ruth Bedoy, Daniel Felipe-Morales, Kimberly Driscoll, Brigitte I. Frohnert, Patricia Gesualdo, Michelle Hoffman, Rachel Karban, Edwin Liu, Jill Norris, Adela Samper-Imaz, Andrea Steck, Kathleen Waugh, Hali Wright, Jorma Toppari, Olli G. Simell, Annika Adamsson, Suvi Ahonen, Jorma Ilonen, Sanna Jokipuu, Tiina Kallio, Leena Karlsson, Miia Kähönen, Mikael Knip, Lea Kovanen, Mirva Koreasalo, Kalle Kurppa, Tiina Latva-aho, Maria Lönnrot, Elina Mäntymäki, Katja Multasuo, Juha Mykkänen, Tiina Niininen, Sari Niinistö, Mia Nyblom, Petra Rajala, Jenna Rautanen, Anne Riikonen, Mika Riikonen, Jenni Rouhiainen, Minna Romo, Tuula Simell, Ville Simell, Maija Sjöberg, Aino Stenius, Maria Leppänen, Sini Vainionpää, Eeva Varjonen, Riitta Veijola, Suvi M. Virtanen, Mari Vähä-Mäkilä, Mari ÅkerlundKatri Lindfors, Jin-Xiong She, Desmond Schatz, Diane Hopkins, Leigh Steed, Jamie Thomas, Janey Adams, Katherine Silvis, Michael Haller, Melissa Gardiner, Richard McIndoe, Ashok Sharma, Joshua Williams, Gabriela Young, Stephen W. Anderson, Laura Jacobsen, Anette G. Ziegler, Andreas Beyerlein, Ezio Bonifacio, Michael Hummel, Sandra Hummel, Kristina Foterek, Nicole Janz, Mathilde Kersting, Annette Knopff, Sibylle Koletzko, Claudia Peplow, Roswith Roth, Marlon Scholz, Joanna Stock, Katharina Warncke, Lorena Wendel, Christiane Winkler, Åke Lernmark, Daniel Agardh, Carin Andrén Aronsson, Maria Ask, Jenny Bremer, Ulla-Marie Carlsson, Corrado Cilio, Emelie Ericson-Hallström, Lina Fransson, Thomas Gard, Joanna Gerardsson, Rasmus Bennet, Monica Hansen, Gertie Hansson, Susanne Hyberg, Fredrik Johansen, Berglind Jonsdottir, Helena Elding Larsson, Marielle Lindström, Markus Lundgren, Maria Månsson-Martinez, Maria Markan, Jessica Melin, Zeliha Mestan, Karin Ottosson, Kobra Rahmati, Anita Ramelius, Falastin Salami, Sara Sibthorpe, Birgitta Sjöberg, Ulrica Swartling, Evelyn Tekum Amboh, Carina Törn, Anne Wallin, Åsa Wimar, Sofie Åberg, William A. Hagopian, Michael Killian, Claire Cowen Crouch, Jennifer Skidmore, Josephine Carson, Maria Dalzell, Kayleen Dunson, Rachel Hervey, Corbin Johnson, Rachel Lyons, Arlene Meyer, Denise Mulenga, Alexander Tarr, Morgan Uland, John Willis, Dorothy Becker, Margaret Franciscus, MaryEllen Dalmagro-Elias Smith, Ashi Daftary, Mary Beth Klein, Chrystal Yates, Jeffrey P. Krischer, Michael Abbondondolo, Sarah Austin-Gonzalez, Maryouri Avendano, Sandra Baethke, Rasheedah Brown, Brant Burkhardt, Martha Butterworth, Joanna Clasen, David Cuthbertson, Christopher Eberhard, Steven Fiske, Dena Garcia, Jennifer Garmeson, Veena Gowda, Kathleen Heyman, Francisco Perez Laras, Hye-Seung Lee, Shu Liu, Xiang Liu, Kristian Lynch, Jamie Malloy, Cristina McCarthy, Steven Meulemans, Hemang Parikh, Chris Shaffer, Laura Smith, Susan Smith, Noah Sulman, Roy Tamura, Ulla Uusitalo, Kendra Vehik, Ponni Vijayakandipan, Keith Wood, Jimin Yang, Beena Akolkar, Liping Yu, Dongmei Miao, Polly Bingley, Alistair Williams, Kyla Chandler, Saba Rokni, Claire Williams, Rebecca Wyatt, Gifty George, Sian Grace, Henry Erlich, Steven J. Mack, Anna Lisa Fear, Sandra Ke, Niveen Mulholland, Stephen S. Rich, Wei-Min Chen, Suna Onengut-Gumuscu, Emily Farber, Rebecca Roche Pickin, Jordan Davis, Dan Gallo, Jessica Bonnie, Paul Campolieto, Kasia Bourcier, Thomas Briese, Suzanne Bennett Johnson, Eric Triplett

**Affiliations:** 1 Department of Clinical Sciences, Diabetes and Celiac disease unit, Lund University, Clinical Research Centre, Jan Waldenströms gata 35, 205 02 Malmö, Sweden; 2 Center for Biotechnology and Genomic Medicine, Medical College of Georgia, Augusta University, Augusta, GA USA; 3 Health Informatics Institute, Morsani College of Medicine, University of South Florida, Tampa, FL USA; 4 Barbara Davis Center for Childhood Diabetes, University of Colorado, Aurora, CO USA; 5 Pacific Northwest Diabetes Research Institute, Seattle, WA USA; 6 Department of Physiology, Institute of Biomedicine, University of Turku, and Department of Pediatrics, Turku University Hospital, Turku, Finland; 7 Institute of Diabetes Research, Helmholtz Zentrum München, and Klinikum rechts der Isar, Technische Universität München, and Forschergruppe Diabetes e.V, Neuherberg, Germany; 8 National Institute of Diabetes & Digestive & Kidney Diseases, Bethesda, MD USA; 9 Department of Pediatrics, University of Florida, Gainesville, FL USA

**Keywords:** Type 1 diabetes, Analgesics, Islet autoimmunity, Prospective studies

## Abstract

**Background:**

The use of analgesic antipyretics (ANAP) in children have long been a matter of controversy. Data on their practical use on an individual level has, however, been scarce. There are indications of possible effects on glucose homeostasis and immune function related to the use of ANAP. The aim of this study was to analyze patterns of analgesic antipyretic use across the clinical centers of The Environmental Determinants of Diabetes in the Young (TEDDY) prospective cohort study and test if ANAP use was a risk factor for islet autoimmunity.

**Methods:**

Data were collected for 8542 children in the first 2.5 years of life. Incidence was analyzed using logistic regression with country and first child status as independent variables. Holm’s procedure was used to adjust for multiplicity of intercountry comparisons. Time to autoantibody seroconversion was analyzed using a Cox proportional hazards model with cumulative analgesic use as primary time dependent covariate of interest. For each categorization, a generalized estimating equation (GEE) approach was used.

**Results:**

Higher prevalence of ANAP use was found in the U.S. (95.7%) and Sweden (94.8%) compared to Finland (78.1%) and Germany (80.2%). First-born children were more commonly given acetaminophen (OR 1.26; 95% CI 1.07, 1.49; *p* = 0.007) but less commonly Non-Steroidal Anti-inflammatory Drugs (NSAID) (OR 0.86; 95% CI 0.78, 0.95; *p* = 0.002). Acetaminophen and NSAID use in the absence of fever and infection was more prevalent in the U.S. (40.4%; 26.3% of doses) compared to Sweden, Finland and Germany (*p* < 0.001).

Acetaminophen or NSAID use before age 2.5 years did not predict development of islet autoimmunity by age 6 years (HR 1.02, 95% CI 0.99-1.09; *p* = 0.27). In a sub-analysis, acetaminophen use in children with fever weakly predicted development of islet autoimmunity by age 3 years (HR 1.05; 95% CI 1.01-1.09; *p* = 0.024).

**Conclusions:**

ANAP use in young children is not a risk factor for seroconversion by age 6 years. Use of ANAP is widespread in young children, and significantly higher in the U.S. compared to other study sites, where use is common also in absence of fever and infection.

**Electronic supplementary material:**

The online version of this article (doi:10.1186/s12887-017-0884-y) contains supplementary material, which is available to authorized users.

## Background

The administration of analgesic-antipyretic (ANAP) medications to children has been discussed in the literature for decades. Surveys of Canadian and American pediatricians reflect the routine use of acetaminophen and non-steroidal anti-inflammatory drugs (NSAID) for childhood fever and discomfort [1, 2]. In the 1980s, the term “fever phobia” was used to describe the parental pressure facing pediatric practitioners to manage fever [3]. Parental misconceptions often lead parents to the inappropriate management of fever in their children [4] and parents report the use of antipyretics even when there was minimal or no fever [5] as parents were frequently concerned with the need to maintain a “normal temperature” in their ill child [6]. Nevertheless, additional studies are needed to support this as evidence-based practice [7, 8]. Acetaminophen and NSAID are used widely in children, but limited data exist regarding patterns of use in countries beyond the United States, United Kingdom, France, and Canada [9].

Notably, acetaminophen has been shown to have effects on glucose homeostasis. High doses have been shown to induce hyperglycemia [10], whereas low and chronic doses can lower blood glucose in animal models [11–13]. Possible effects on asthma risk have also been investigated [14, 15]. NSAIDs have also been shown to lower blood glucose [16, 17], but have additional anti-inflammatory properties that could have an impact on the process leading up to T1D [18].

The Environmental Determinants of Diabetes in the Young (TEDDY) Study is an international, multi-center study designed to identify the environmental triggers of T1D in genetically at-risk children [19]. The aim of the current study was to describe the use of ANAP in the TEDDY study, as well as differences in relation to country, birth order (first child versus a child with older siblings) and fever status. Specifically, we sought to examine if the use of ANAP: (1) is associated with risk for islet autoimmunity (IA), (2) differs between countries, (3) is given preferentially to first-born children.

## Methods

The Environmental Determinants of Diabetes in the Young (TEDDY) is a prospective cohort study funded by the National Institutes of Health with the primary goal to identify environmental causes of type 1 diabetes (T1D). It includes six clinical research centers - three in the US: Colorado, Georgia/Florida, Washington and three in Europe: Finland, Germany, and Sweden. Detailed study design and methods have been previously published [19, 20]. Written informed consents were obtained for all study participants from a parent or primary caretaker for genetic screening and participation in prospective follow-up. The study was approved by local Institutional or Ethics Review Boards (Additional file 1), and is monitored by an External Advisory Board formed by the National Institutes of Health.

### Data collection

The dataset analyzed was the data received by the TEDDY Data Coordinating Center as of December 31, 2014. The total number of subjects enrolled was 8676. Analysis was restricted to confirmed HLA eligible subjects and subjects with medication information in the first 2 years of age. Out of the enrolled subjects, 134 were missing medication data and were excluded from the analysis. Information regarding first child status was missing for 919 subjects who were also excluded, leaving a total of 7623 subjects (Additional file 2).

Study visits were conducted every 3 months with the first visit occurring between 3 and 4.5 months of age. At each visit, interviewers recorded the name, reason, start date and duration of reported medications for the most recent visit interval. Parents were asked to document fever as either “Yes” or “No” for every illness entry in a “TEDDY Book.” The “TEDDY Book” provided written guidance that “Yes” should only be marked for temperature equal to or greater than 38 °C or 101 °F. Approximately 18 months into the study, these choices were expanded to “Yes – measured,” “Yes – not measured,” and “No.” The rationale for this change was to capture all uses of ANAP, even with low-grade fevers.

Each use of an ANAP was defined as an episode. Recorded medications were categorized based on active ingredient. When analyzing specific substances, all medications containing that particular substance were included. Drugs were also defined and grouped as either analgesic or non-steroidal anti-inflammatory (NSAID) (Additional file 3). Episodes were described as associated with infection and/or fever. Infection was defined as either an ICD-10 code indicating Infection (Additional file 4) or an acute illness designated as infectious within a 15 day time period of the medication date [21]. Fever was defined as either an ICD-10 code of fever associated with the medication or an acute illness associated with fever within a 15-day time period of the medication date.

### Islet autoimmunity

Blood samples were drawn every 3 months between 3 and 48 months of age, and every 6 months thereafter, except for autoantibody positive children, who continued with visits every 3 months. Persistent IA was defined as positive antibodies to insulin (IAA), glutamic acid decarboxylase (GAD65), or insulinoma-associated antigen 2 (IA-2), each analyzed by radiobinding assay [22, 23], on at least 2 consecutive study visits. Two central autoantibody laboratories were used; one in the U.S. (Barbara Davis Center for Childhood Diabetes at the University of Colorado) and one in Europe (University of Bristol). All positive islet autoantibodies and 5% of negative islet autoantibodies were confirmed in both central autoantibody laboratories. Both laboratories have previously demonstrated high sensitivity, specificity [24] and concordance. Positive results in the child that were deemed to be due to maternal IgG transmission were excluded from the IA-positive group.

### Statistical methods

A Cox proportional hazards model was used to assess the impact of ANAP use in the first 90, 180, 365 days of age and 2.5 years of age in the risk of positive autoantibodies through 6 years of age. The number of infections early in life was included as a time dependent covariate [25]. Country was included as a stratification factor in the proportional hazards analyses. Additional covariates included in the model were first-degree relative [26], HLA [27], gender, ever breastfed [28, 29], probiotic use prior to 3 months of age [30], and eight different previously identified single nucleotide polymorphisms [31]. The primary variable of interest was cumulative ANAP use through 2.5 years of life as a time dependent covariate. Included covariates can be seen in Table 1.

**Table 1:** Covariates included in the Cox proportionate hazards analysis of time to persistent confirmed autoantibody positivity

Fixed Covariates		Hazard Ratio (95% CI)	Wald test p-value^a^
First-Degree Relative (Ref = No)		2.51 (2.06, 3.30)	<0.001
HLA (Ref = DR3/DR4)			
	DR4/DR4	0.69 (0.54, 0.88)	0.003
	DR4/DR8	0.70 (0.54, 0.91)	0.008
	DR3/DR3	0.46 (0.35, 0.60)	<0.001
	All Others	0.46 (0.29, 0.72)	<0.001
Gender (Ref = male)		0.77 (0.65, 0.92)	0.003
SNP			
	RS1004446_a	0.84 (0.74, 0.96)	0.010
	RS10517086_a	1.14 (1.00, 1.31)	0.050
	RS12708716_g	0.87 (0.76, 0.99)	0.034
	RS2292239_a	1.24 (1.09, 1.41)	<0.001
	RS2476601_a	1.55 (1.31, 1.83)	<0.001
	RS2816316_c	1.07 (0.91, 1.25)	0.429
	RS3184504_a	1.33 (1.17, 1.50)	<0.001
	RS4948088_a	0.74 (0.53, 1.04)	0.086
Ever Breastfed (Ref = No)		1.96 (1.01, 3.81)	0.042
Probiotics <3 Mo Age (Ref = No)		0.72 (0.55, 0.94)	0.015
Time Dependent Covariates			
Cumulative Number of Infections		1.02 (0.99, 1.03)	0.407
Cumulative Weeks Analgesic Use		1.02 (0.99, 1.04)	0.269

Number of persistent confirmed cases = 511

^a^Ho: Hazard Ratio = 1

The statistical analysis for the number of episodes per year and duration per year excluded subjects for which the first child status was missing. Subjects with a missing duration for a specific analgesic were excluded from the analysis for that analgesic. The statistical analysis of total duration per year was based on log-transformed data to better satisfy the assumptions of the linear models.

Subject incidence was analyzed using logistic regression with country and first child status as independent variables in the model. In both the binary and continuous analyses, pairwise comparisons between countries were conducted using Holm’s procedure to adjust for the multiplicity of comparisons. Each specific episode of ANAP usage was classified by concurrent fever (yes/no) or infection (yes/no). Episodes were categorized as associated with Fever, Infection, both Fever and Infection, or neither fever nor infection. For each categorization, a generalized estimating equation (GEE) was used for analysis with country and first child as independent variables in the model. An ignorable working matrix was assumed for the GEE analysis with the empirical sandwich estimate used for the standard errors. Pair-wise comparisons across countries were conducted using Holm’s procedure from the GEE analyses. Analyses on the episode level excluded subjects who reported no episodes.

Statistical analysis was performed using SAS version 9.3 (SAS Institute Inc., Cary, NC, U.S.A).

## Results

### Use of ANAP below the age of 2.5 years

**Fig. 1:**
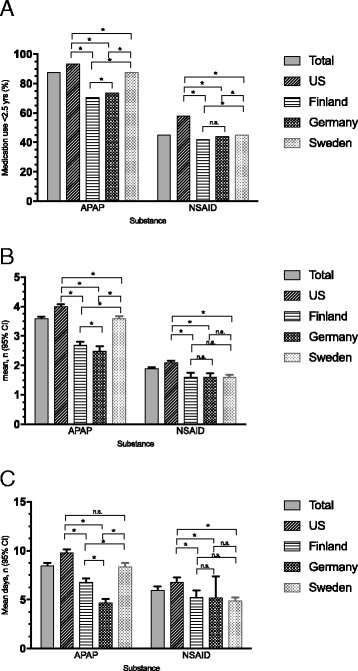
Use of ANAP below 2,5 years of age. *:*p* < 0.05, n.s.: non significant. All significances corrected for multiple comparisons using Holm procedure. **a** Prevalence of analgesic/antipyretic use. **b** Treatment episodes per year. **c** Duration of medication use per year

The use of both acetaminophen and NSAIDs were very common in the study population. In the total cohort, 87.8% of children reported the use of acetaminophen and 45.4% of NSAIDs before the age of 2.5 years. The mean number of treatment episodes per year was 3.6 ± 2.1 and mean duration of treatment 8.5 ± 10.8 days per year in the total cohort (Fig. 1a–c).

### Acetaminophen use

Swedish parents reported a significantly higher prevalence of acetaminophen use (94.5%), followed by U.S. (93.7%), Finnish (73.9%), and German parents (70.1%). Prevalence differed between all countries (Finland vs. Germany: *p* = 0.035, all other *p* < 0.001). U.S. parents reported the highest number of treatment episodes per year and highest total duration of treatment per year (mean 4.0 ± 2.3 episodes; mean 9.8 ± 13.5 days), followed by Swedish (mean 3.6 ± 2.1 episodes; mean 8.4 ± 8.6 days), Finnish (mean 2.7 ± 1.9 episodes; mean 6.8 ± 6.4 days), and German parents (mean 2.5 ± 1.6 episodes; mean 4.7 ± 3.7 days). All country differences, according to number of treatment episodes, were statistically significant (Finland vs. Germany: *p* = 0.014, all others *p* < 0.001) (Fig. 1). Children born as the first child in the family had more often been given acetaminophen during their first 2.5 years of life compared to children with older siblings (OR 1.26; 95% CI 1.07, 1.49; *p* = 0.007). The number of episodes of treatment with acetaminophen was also higher (difference in least square means 0.15; 95% CI 0.05, 9.24; *p* = 0.003). No difference could be seen regarding the number of days treated (difference in least square means 0.11; 95% CI -0.38, 0.60; *p* = 0.111).

### NSAID use

The highest prevalence of NSAID use was reported by U.S. parents (58.3%), followed by German (44.1%), Finnish (42.3%), and Swedish parents (29.0%). All country differences, except between Finland and Germany, were statistically significant (Finland vs. Germany: *p* = 0.177, all others *p* < 0.001). U.S. parents reported the highest number of treatment episodes with NSAID per year (mean 2.1 ± 1.4 episodes; mean 6.8 ± 11.2 days), followed by Swedish, Finnish, and German parents (mean 1.6 ± 1.2; mean 1.6 ± 1.2; mean 1.6 ± 1.1) Total duration of treatment was highest in the U.S. (mean 6.8 ± 11.2 days), followed by Finland (mean 5.3 ± 9.3 days), Germany (mean 5.2 ± 17.2 days), and Sweden (mean 4.9 ± 4.8 days). Both the mean number of treatment episodes and mean total duration of treatment were significantly higher in the U.S. compared to the other countries (*p* < 0.001). No other significant country differences could be seen (Fig. 1). The prevalence of NSAID use during the first 2.5 years of life were lower in first-born children (OR 0.86; 95% CI 0.78, 0.95; *p* = 0.002) and they were also treated fewer times (difference in least square means −0.14; 95% CI -0.22, −0.05; *p* = 0.001). No differences could be seen regarding the number of days treated (difference in least square means −0.22; 95% CI -0.92, 0.48; *p* = 0.143).

### Differences in use for febrile/infectious episodes and noninfectious use

In the total cohort, 74.1% of acetaminophen use and 82.0% of NSAID use was given in conjunction with either fever, infection or both, with 43.8% acetaminophen use and 51.0% NSAID episodes being combined fever and infection. U.S. parents reported a significantly higher proportion of doses given without fever or infection for both acetaminophen (40.4%) and NSAID (26.3%) compared to the other three countries (*p* < 0.001). Acetaminophen use in feverish infectious episodes had the highest proportion among German and Swedish children (68.5% and 63.2%;), followed by Finland with 57.9% and the U.S. with 23.5% (all *p*-values for differences between countries were *p* < 0.001, except between Germany and Swedenwas *p* = 0.003).

**Fig. 2:**
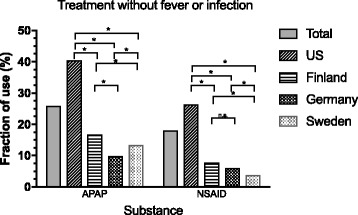
Fraction of treatment without fever and infection. *:*p* < 0.05, n.s.: non-significant. All significances corrected for multiple comparisons using Holm procedure

For NSAID use without fever or infection, the U.S. parents reported the highest proportion (26.3%), followed by Finland (7.7%), Germany (5.9%), and Sweden (3.7%) (difference between Finland and Sweden *p* = 0.006; between Germany and Sweden *p* = 0.01; all others *p* < 0.001) (Fig. 2, Table 2).

**Table 2:** Summary of treatment episodes associated with fever and/or infection

			US	Finland	Germany	Sweden	Total Cohort	Country differences					
								U-F	U-G	U-S	F-G	F-S	G-S
Total episodes		n	25,340	7427	2091	17,285	52,643						
Acetaminophen	Fever and infection	n	4272	2907	983	9224	17,386	<0.001	<0.001	<0.001	<0.001	<0.001	0.003
	Fever or infection		6557	1277	311	3421	12,066						
	No fever and no infection	n	7348	841	141	1953	10,283	<0.001	<0.001	<0.001	<0.001	0.006	0.01
NSAID	Fever and infection	n	2680	1273	477	1572	6002	<0.001	<0.001	<0.001	<0.001	<0.001	0.024
	Fever or infection	n	2472	717	125	330	3644						
	No fever and no infection	n	1841	167	38	74	2120	<0.001	<0.001	<0.001	0.174	<0.001	0.041

Country differences: U = US, G = Germany, F = Finland, S = Sweden, Country differences described as *p*-value for difference between the respective countries

### Islet autoimmunity

Hazard ratios for islet autoimmunity were estimated for cumulative use of acetaminophen and NSAID with or without concomitant fever and for a joint variable of cumulative total ANAP use with or without fever. A significant hazard was only found for use of acetaminophen in the presence of fever for islet autoimmunity at age 3 years (HR 1.05; 95% CI 1.01-1.09; *p* = 0.024). The hazard was not significant for islet autoimmunity at 6 years of age (*p* = 0.193).

Separate analysis of exposure before 90, 180 and 365 days of life found a significant hazard for seroconversion at age 3 years (HR 1.06; 95% CI 1.00-1.12; *p* = 0.011) for use of acetaminophen with concurrent fever before 1 year of age, but not before 90 or 180 days of life (*p* = 0.91 and *p* = 0.54, respectively). No other significant hazards could be seen for treatment with acetaminophen or NSAID in the presence or absence of fever (Table 3).

**Table 3:** Hazard ratios for seroconversion to persistent islet autoimmunity at 3 and 6 years of age for analgesic variables of interest

Analgesic Variable	Exposed subjects n (%)	3 Year Analysis	6 Year Analysis
		398 antibody + subjects	511 antibody + subjects
		HR (95% CI) *p*-value	HR (95% CI) *p*-value
Acetaminophen, any exposure	7496 (87.8%)	1.01 (0.97, 1.05) 0.603	1.01 (0.98, 1.05) 0.576
Acetaminophen with fever + infection	5179 (60.6%)	1.05 (1.01, 1.09) 0.022	1.03 (0.99, 1.08) 0.189
Exposed <90 days of life	220 (2.6%)	0.97 (0.59, 1.61) 0.914	1.00 (0.68, 1.46) 0.986
Exposed <180 days of life	1519 (17.8%)	1.07 (0.87, 1.32) 0.542	1.06 (0.88, 1.27) 0.527
Exposed <365 days of life	3795 (44.4%)	1.06 (1.00, 1.12) 0.011	1.05 (0.99, 1.11) 0.101
Acetaminophen without fever or infection	5941 (69.6%)	1.02 (0.98, 1.06) 0.346	1.01 (0.96, 1.05) 0.769
Exposed <90 days of life	4016 (47.0%)	1.00 (0.90, 1.11) 0.994	0.99 (0.85, 1.14) 0.837
Exposed <180 days of life	4597 (53.8%)	0.98 (0.84, 1.14) 0.814	0.97 (0.84, 1.13) 0.729
Exposed <365 days of life	5310 (62.2%)	1.03 (0.99, 1.06) 0.114	1.02 (0.99, 1.06) 0.228
NSAID, any exposure	3874 (45.4%)	1.01 (0.97, 1.05) 0.753	1.01 (0.97, 1.04) 0.763
NSAID with fever + infection	2652 (31.0%)	1.01 (0.97, 1.05) 0.673	1.01 (0.98, 1.05) 0.459
Exposed <90 days of life	22 (0.3%)	1.01 (0.92, 1.10) 0.897	1.00 (0.92, 1.09) 0.960
Exposed <180 days of life	223 (2.6%)	0.99 (0.88, 1.11) 0.822	1.00 (0.94, 1.07) 0.927
Exposed <365 days of life	1318 (15.4%)	1.01 (0.97, 1.06) 0.530	1.02 (0.98, 1.05) 0.378
NSAID without fever or infection	1955 (22.9%)	1.00 (0.96, 1.05) 0.856	1.01 (0.97, 1.05) 0.623
Exposed <90 days of life	222 (2.6%)	1.00 (0.88, 1.12) 0.943	0.99 (0.88, 1.12) 0.874
Exposed <180 days of life	458 (5.4%)	0.99 (0.88, 1.11) 0.815	1.00 (0.94, 1.07) 0.956
Exposed <365 days of life	1120 (13.1%)	1.01 (0.96, 1.06) 0.824	1.01 (0.97, 1.05) 0.638
Any analgesic, any exposure	7744 (91%)	1.02 (0.99, 1.05) 0.130	1.02 (0.99, 1.04) 0.267
Any analgesic with fever + infection	5699 (67%)	1.06 (0.97, 1.15) 0.219	1.02 (0.94, 1.10) 0.667

Each hazard ratio is calculated from a Cox proportional hazards model with the analgesic variable and covariates indicated in text

## Discussion

In this study, we investigated the use of ANAP in children below the age of 2.5 years, and the impact of such use on the development of islet autoimmunity before 6 years of age in the large, longitudinal, international TEDDY cohort.

The widespread use of acetaminophen among young children has been of interest due not only to possible side effects, but also possible immunological effects. Several papers have investigated the impact on immune response and the development of autoimmunity [11, 12, 32, 33]. The data on childhood asthma is conflicting, with some studies showing an increased risk and others showing none [14, 15, 34]. The use of prophylactic acetaminophen in conjunction with childhood vaccinations has also shown possible effects on antibody responses [1, 7, 35]. In this study, we found a significant but weak increased hazard ratio associated with the use of acetaminophen and concomitant fever before the age of 2.5 years and persistent confirmed islet autoimmunity at age 3 years. However, this effect was not seen with islet autoimmunity at age 6 years or if acetaminophen was used for other reasons. It is therefore unlikely, although possible, that this is a true effect. The type of infection causing the fever may be a confounding factor. No such effect was seen with the use of NSAIDs or the combination of acetaminophen and NSAIDs, either when given with or without fever.

The use of acetaminophen and NSAIDs for the treatment of children has been previously described from a medical standpoint [36, 37]. On the other hand, very little has been described regarding practical use in the pediatric population. This analysis within a large international cohort provides some of the first data regarding pediatric use of ANAP. As expected,the majority of treatment episodes for this young cohort were in conjunction with fever and/or infection. It is worth mentioning that there are significant differences between the TEDDY countries regarding the use of both acetaminophen and NSAIDs. The U.S. stands out for both greater prevalence of use and greater number of episodes of treatment per year, followed closely by Sweden in regards to acetaminophen use. U.S. parents were also just as likely to report using these medications during episodes associated with infection than non-infectious episodes. Additionally, they were more likely to use ANAP when there was no associated fever. It may be a common practice of American physicians to prescribe a combination therapy approach to the use of analgesics and antipyretics forthe sustained management of fever. However, parents may then assume that even prophylactic use should be a combination therapy.

We also found that first-born children were preferentially given acetaminophen, both in prevalence of use and in the higher number of treatment episodes than for their younger siblings. The inverse relationship was observed for NSAID use, in which both the prevalence and number of treatment episodes were lower for first-born children. We can only speculate on the possible rationale behind this finding since, to our knowledge, no earlier study has presented similar data. It is possible that acetaminophen is perceived by first-time parents as a better tolerated treatmentthan NSAIDS, a perception that fades by the time younger siblings require treatment.

Country-specific differences in the use of analgesics may be culturally influenced. The lower incidence of use of all standard analgesics in Germany could reflect the prevalence of Complimentary Alternative Medicine (CAM) in this country. According to a cross-sectional survey of German physicians in 2007, more than two-thirds of patients in Germany use CAM provided either by physicians or non-medical practitioners (“Heilpraktiker”) [38]. In 2007, only 40% of adults in the U.S. had used CAM therapy in the past 12 months. Children in the U.S. whose parents used CAM were almost five times as likely (23.9%) to use CAM than children whose parent did not use CAM (5.1%) [39]. The reasons underlying greater use of acetaminophen among Swedish parents is more unclear but may be the result of acetaminophen being widely available and perceived as safe and effective.

The TEDDY study is one of the largest longitudinal pediatric cohorts studied. The data analyzed herein has been collected from parent reports given in writing and after discussion with a TEDDY nurse. For participating children, missing data is uncommon. Follow-up is continuous from age 3 months which minimizes recall bias. The possibility to adjust for confounding factors in the statistical analysis is great due to the availability of comprehensive data on the child’s living conditions. All previously described risk factors for T1D and islet autoimmunity are also entered into the statistical analysis of the effect of analgesics on islet autoimmunity.

The limitations of this study include our reliance on parent-reported symptoms and dosages of ANAP. The size of the cohort also makes it challenging to confirm diagnoses and treatment plans via patient records. Notably, most of the reported infections were presumed to be viral infections for which no medical advice had been sought. In addition, the widespread use of ANAP in this age group poses a significant statistical problem since the exposed group widely surpasses the non-exposed group. Even with the large sample size, the resulting correlation between IA and acetaminophen in combination with fever must therefore be interpreted with caution.

## Conclusions

In conclusion, the use of ANAP to treat fever and infection is widespread in the TEDDY cohort but shows significant differences depending on study site. The prevalence of use of both acetaminophen and NSAIDs are highest in the U.S. and lowest in Finland and Germany. Use of both NSAIDs and acetaminophen for non-infectious purposes are significantly more common among children in the U.S. compared to those in Europe. No convincing effect on risk for autoimmunity can be seen in the analysis except for a small effect by acetaminophen in combination with fever, and then only for autoimmunity at 3 years of age.

## Additional files

Additional file 1: Ethical review boards and Committees granting ethical approval to the TEDDY study. Listing of all ethical review boards and committes that has granted approval to the TEDDY study for the respective sites. (DOCX 59 kb)

Additional file 2: Characteristics of the subjects used in analysis for analgesic use and islet cell autoimmunity. A table the prevalence of covariates in the present analysis including HLA-DQ genotype, Gender, first degree relative, breastfeeding, probiotic use and presence of included single nucleotide polymorphisms. (DOCX 106 kb)

Additional file 3: List of Drugs defined as analgesics used in first 2.5 years in the TEDDY study. List of all included drugs, recorded to having been given to TEDDY children before age 2.5 years and classified as analgesics. Drugs classified as NSAID’s are marked with a star (*). (DOCX 30 kb)

Additional file 4: List of all ICD-10 codes, recorded among TEDDY children before age 2.5 years, and classified as an infection. (DOCX 72 kb)

## Abbreviations

ANAP: Analgesic-Antipyretic
CAM: Complimentary alternative medicine
GEE: Generalized estimating eq.
IA: Islet Autoimmunity
NSAID: Non-steroidal anti-inflammatory drugs
T1D: Type 1 Diabetes
TEDDY: The environmental determinants of diabetes in the young study
