# Supplementary material for: Analgesic antipyretic use among young children in the TEDDY study: no association with islet autoimmunity
Source: BMC Pediatr. 2017 May 16;17:127. doi: 10.1186/s12887-017-0884-y (PMC5434629; doi:10.1186/s12887-017-0884-y)
Supplement: Supplementary file 1 — Ethical review boards and Committees granting ethical approval to the TEDDY study. Listing of all ethical review boards and committes that has granted approval to the TEDDY study for the respective sites. (DOCX 59 kb) [file 12887_2017_884_MOESM1_ESM.docx]

**Appendix A. Ethical review boards and Committees granting ethical approval to the TEDDY study.**

Colorado: Colorado Multiple Institutional Review Board

Georgia: Medical College of Georgia Human Assurance Committee (2004-2010) Georgia Health Sciences University Human Assurance Committee (2011-2012) Georgia Regents University Institutional Review Board (2013-2015) Augusta University Institutional Review Board (2015-present)

Florida: University of Florida Health Center Institutional Review Board

Washington: Washington State Institutional Review Board (2004-2012)
Western Institutional Review Board (2013-present)

Finland: Ethics Committee of the Hospital District of Southwest Finland

Germany: Bayerischen Landesärztekammer (Bavarian Medical Association) Ethics Committee

Sweden: Regional Ethics Board in Lund, Section 2 (2004-2012)
Lund University Committee for Continuing Ethical Review (2013-present)
