# Supplementary material for: Analgesic antipyretic use among young children in the TEDDY study: no association with islet autoimmunity
Source: BMC Pediatr. 2017 May 16;17:127. doi: 10.1186/s12887-017-0884-y (PMC5434629; doi:10.1186/s12887-017-0884-y)
Supplement: Supplementary file 2 — Characteristics of the subjects used in analysis for analgesic use and islet cell autoimmunity. A table the prevalence of covariates in the present analysis including HLA-DQ genotype, Gender, first degree relative, breastfeeding, probiotic use and presence of included single nucleotide polymorphisms. (DOCX 106 kb) [file 12887_2017_884_MOESM2_ESM.docx]

**Appendix B. Characteristics of the subjects used in analysis for analgesic use and islet cell autoimmunity**.

|  | Overall | United States | Finland | Germany | Sweden |
| --- | --- | --- | --- | --- | --- |
|  | N=6894 | N=2838 | N=1582 | N=425 | N=2049 |
| HLA |  |  |  |  |  |
| DR3/DR4 | 2684 (39%) | 1133 (40%) | 525 (33%) | 164 (39%) | 862 (42%) |
| DR4/DR4 | 1358 (20%) | 582 (21%) | 256 (16%) | 70 (16%) | 450 (22%) |
| DR4/DR8 | 1192 (17%) | 382 (13%) | 508 (32%) | 39 (9%) | 263 (13%) |
| DR3/DR3 | 1428 (21%) | 665 (23%) | 231 (15%) | 89 (21%) | 443 (22%) |
| All Others | 232 (3%) | 76 (3%) | 62 (4%) | 63 (15%) | 31 (2%) |
| Gender: Male | 3513 (51%) | 1458 (51%) | 805 (51%) | 218 (51%) | 1032 (50%) |
| 1^st^ Degree Relative^1^ | 789 (11%) | 325 (11%) | 143 (9%) | 173 (41%) | 148 (7%) |
| Ever Breastfed^2^ | 6640 (96%) | 2646 (93%) | 1573 (99%) | 397 (93%) | 2024 (99%) |
| Probiotic Use^3^ | 1044 (15%) | 75 (3%) | 609 (39%) | 109 (26%) | 251 (12%) |
| SNP rs1004446^4^ |  |  |  |  |  |
| 0 | 2766 (40%) | 1151 (41%) | 663 (42%) | 174 (41%) | 778 (38%) |
| 1 | 3180 (46%) | 1302 (46%) | 728 (46%) | 196 (46%) | 954 (47%) |
| 2 | 948 (14%) | 385 (14%) | 191 (12%) | 55 (13%) | 317 (15%) |
| SNP rs10517086 ^4^ |  |  |  |  |  |
| 0 | 3523 (51%) | 1476 (52%) | 790 (50%) | 191 (45%) | 1066 (52%) |
| 1 | 2826 (41%) | 1144 (40%) | 672 (42%) | 203 (48%) | 807 (39%) |
| 2 | 545 (8%) | 218 (8%) | 120 (8%) | 31 (7%) | 176 (9%) |
| SNP rs12708716^4^ |  |  |  |  |  |
| 0 | 3042 (44%) | 1248 (44%) | 724 (46%) | 174 (41%) | 896 (44%) |
| 1 | 3053 (44%) | 1265 (45%) | 680 (43%) | 191 (45%) | 917 (45%) |
| 2 | 799 (12%) | 325 (11%) | 178 (11%) | 60 (14%) | 236 (12%) |
| SNP rs2292239^4^ |  |  |  |  |  |
| 0 | 3154 (46%) | 1363 (48%) | 743 (47%) | 196 (46%) | 852 (42%) |
| 1 | 3045 (44%) | 1228 (43%) | 679 (43%) | 181 (43%) | 957 (47%) |
| 2 | 695 (10%) | 247 (9%) | 160 (10%) | 48 (11%) | 240 (12%) |
| SNP rs2476601^4^ |  |  |  |  |  |
| 0 | 5472 (79%) | 2348 (83%) | 1158 (73%) | 327 (77%) | 1639 (80%) |
| 1 | 1331 (19%) | 465 (16%) | 389 (29%) | 90 (21%) | 387 (19%) |
| 2 | 91 (1%) | 25 (1%) | 35 (2%) | 8 (2%) | 23 (1%) |
| SNP rs2816316^4^ |  |  |  |  |  |
| 0 | 4612 (67%) | 1839 (65%) | 1145 (72%) | 280 (66%) | 1348 (66%) |
| 1 | 2049 (30%) | 886 (31%) | 399 (25%) | 128 (30%) | 636 (31%) |
| 2 | 233 (3%) | 113 (4%) | 38 (2%) | 17 (4%) | 65 (3%) |
| SNP rs3184504^4^ |  |  |  |  |  |
| 0 | 2141 (31%) | 959 (34%) | 545 (34%) | 94 (22%) | 543 (27%) |
| 1 | 3372 (49%) | 1332 (47%) | 767 (48%) | 223 (52%) | 1050 (51%) |
| 2 | 1381 (20%) | 547 (19%) | 270 (17%) | 108 (25%) | 456 (22%) |
| SNP rs4948088^4^ |  |  |  |  |  |
| 0 | 6283 (91%) | 2561 (90%) | 1490 (94%) | 390 (92%) | 1842 (90%) |
| 1 | 590 (9%) | 268 (9%) | 89 (6%) | 33 (8%) | 200 (10%) |
| 2 | 21 (<1%) | 9 (<1%) | 3 (<1%) | 2 (<1%) | 7 (<1%) |

*1: First-degree relative with type 1 diabetes, 2: Breastfed at any point during early life, 3: Probiotic use prior to 1 months of age, 4: Number of minor alleles*
