# Supplementary material for: Analgesic antipyretic use among young children in the TEDDY study: no association with islet autoimmunity
Source: BMC Pediatr. 2017 May 16;17:127. doi: 10.1186/s12887-017-0884-y (PMC5434629; doi:10.1186/s12887-017-0884-y)
Supplement: Supplementary file 3 — List of Drugs defined as analgesics used in first 2.5 years in the TEDDY study. List of all included drugs, recorded to having been given to TEDDY children before age 2.5 years and classified as analgesics. Drugs classified as NSAID’s are marked with a star (*). (DOCX 30 kb) [file 12887_2017_884_MOESM3_ESM.docx]

**Appendix C: List of Drugs defined as analgesics used in first 2.5 years in the TEDDY study.** * Indicates those medications considered NSAIDs

MED00001 Ibuprofen *

MED00010 Acetaminophen

MED00011 Acetaminophen + Dextromethorphan Hydrobromide + Pseudoephedrine Hydrochloride

MED00021 Acetaminophen + Hydrocodone

MED00022 Acetaminophen + Oxycodone

MED00031 Aspirin *

MED00052 Celecoxib *

MED00053 Acetaminophen + Pseudoephedrine

MED00059 Acetaminophen + Codeine

MED00067 Acetaminophen + Dextromethorphan Hydrobromide + Pseudoephedrine Hydrochloride + Chlorpheniramine

MED00068 Acetaminophen + Dextromethorphan Hydrobromide + Pseudoephedrine Hydrochloride + Guaifenesin

MED00069 Acetaminophen + Pseudoephedrine Hydrochloride + Diphenhydramine Hydrochloride

MED00086 Hydrocodone

MED00094 Oxycodone

MED00122 Acetaminophen + Dextromethorphan Hydrobromide + Phenylephrine Hydrochloride

MED00128 Promethazine + Codeine

MED00162 Antipyrine + Benzocaine

MED00173 Fentanyl

MED00175 Carbamazepine

MED00184 Morphine

MED00200 Naproxen *

MED00216 Diclofenac *

MED00228 Sulfasalazine

MED00230 Acetaminophen + Doxylamine

MED00232 Ethylmorphine + Acetanilide + Ephedrine

MED00239 Hydromorphone

MED00244 Acetaminophen + Aspirin *

MED00251 Hydrocodone + Phenylephrine + Chlorpheniramine

MED00263 Acetaminophen +

Propoxyphene

MED00274 Ethylmorphine

MED00284 Codeine

MED00297 Ibuprofen + Pseudoephedrine *

MED00310 Acetaminophen + Dextromethorphan + Pseudoephedrine

MED00329 Acetaminophen + Dextromethorphan + Doxylamine

MED00335 Acetaminophen + Pseudoephedrine

MED00373 Ketoprofen *

MED00409 Benzophenone + Dipyrone

MED00434 Chlorpheniramine + Pseudoephedrine + dihydrocodeine

MED00449 Pimecrolimus *

MED00451 olopatadine

MED00470 Acetaminophen + Chlorpheniramine + Dextromethorphan + Phenylephrine

MED00480 Antipyrine + Procaine

MED00501 Acetaminophen + Phenylephrine

MED00511 Codeine + Guaifenesin

MED00520 Meperidine

MED00527 Acetaminophen + Dextromethorphan + Pseudoephedrine

MED00555 Phenylephrine + Hydrocodone + Guaifenesin

MED00560 Acetaminophen + Chlorpheniramine + Dextromethorphan + Hydrocodone + Ibuprofen + Methscopolamine + Phenylephrine + Pseudoephedrine + Pyrilamine + dihydrocodeine + Brompheniramine + Diphenhydramine + Guaifenesin + Pheniramine + Phenylpropanolamine + Scopolamine + carbetapentane + carbinoxamine + dexbrompheniramine + dexchlorpheniramine + potassium guaiacolsulfonate

MED00573 Acetaminophen + Diphenhydramine + Phenylephrine

MED00597 Ephedrine + Diphenhydramine + Codeine + Cocillana Liquid Extract

MED00603 Acetaminophen + Chlorpheniramine + Dextromethorphan

MED00606 Gabapentin

MED00612 Nitrous Oxide

MED00632 Acetaminophen + Chlorpheniramine + Phenylephrine

MED00641 Diphenhydramine + Hydrocodone + Phenylephrine

MED00678 Chlorpheniramine + hydrocodone + pseudoephedrine

MED00705 Ketamine

MED00751 Acetaminophen + Diphenhydramine

MED00755 Guaifenesin + Hydrocodone

MED00768 Codeine + Methscopolamine + Morphine + Noscapine + Papaverine

MED00784 Acetaminophen + Pheniramine + Phenylephrine

MED00800 Acetaminophen + Dextromethorphan + Doxylamine + Pseudoephedrine

MED00803 Acetaminophen + Dextromethorphan

MED00804 Ketorolac *

MED00822 Tramadol

MED00828 Codeine + Guaifenesin + Ammonium Chloride + Thyme

MED00847 Clonidine

MED00867 Piroxicam *

MED00878 Acetaminophen + Dextromethorphan + Guaifenesin + Phenylephrine

MED00886 Infliximab

MED00894 Alfentanil

MED00911 Dexmedetomidine

MED00928 Dipyrone *

MED00937 Ethylmorphine + Ephedrine

MED00946 Antipyrine + Caffeine

MED00955 Naproxen + Acetaminophen (paracetamol)

MED01010 Indomethacin *

MED01022 Acetaminophen + Dextromethorphan Hydrobromide + Phenylephrine Hydrochloride

MED01040 Hydrocodone + Homatropine

MED01066 Dihydrocodeine

MED01071 Dexbrompheniramine + Phenylephrine + Hydrocodone

MED01075 Pirinitramide

MED01088 Dipyrone + Phenylephrine + Chlorpheniramine

MED01119 Meloxicam *

MED01125 Fluorouracil + Salicylic Acid + Dimethyl Sulfoxide

MED01174 Amantadine

MED01177 Clonidine Hydrochlorid
