# Supplementary material for: Analgesic antipyretic use among young children in the TEDDY study: no association with islet autoimmunity
Source: BMC Pediatr. 2017 May 16;17:127. doi: 10.1186/s12887-017-0884-y (PMC5434629; doi:10.1186/s12887-017-0884-y)
Supplement: Supplementary file 4 — List of all ICD-10 codes, recorded among TEDDY children before age 2.5 years, and classified as an infection. (DOCX 72 kb) [file 12887_2017_884_MOESM4_ESM.docx]

**Appendix D: ICD-10 codes classified as Infection**

A09 Diarrhea and gastroenteritis of presumed infectious origin

A37.9 Whooping cough, unspecified

A49.9 Bacterial infection, unspecified

B08.2 Exanthema subitum [sixth disease ]

B95.1 Streptococcus,group B,as the cause of diseases classified elsewhere

B96.5 Pseudomonas (aeruginosa)(mallei)(pseudomallei)as the cause of diseases classified elsewhere

B97.4 Respiratory syncytial virus as the cause of diseases classified to other chapters

H10 Conjunctivitis

H10.3 Acute conjunctivitis, unspecified

H10.9 Conjunctivitis, unspecified

H66.9 Otitis media, unspecified

J00 Acute nasopharyngitis [common cold]

J01.9 Acute sinusitis, unspecified

J02.9 Acute pharyngitis, unspecified

J03.9 Acute tonsillitis, unspecified

J06.9 Acute upper respiratory infection, unspecified

J12.1 Respiratory syncytial virus pneumonia

J18.9 Pneumonia, unspecified

J20 Acute bronchitis

J20.9 Acute bronchitis, unspecified

J21 Acute bronchiolitis

J21.9 Acute bronchiolitis, unspecified

L01.0 Impetigo [any organism] [any site]

L02.9 Cutaneous abscess, furuncle, and carbuncle, unspecified

L03.0 Cellulitis of finger and toe

L03.9 Cellulitis, unspecified

N10 Acute tubulo-interstitial nephritis

N30.9 Cystitis, unspecified

N39.0 Urinary tract infection, site not specified

P36.9 Bacterial sepsis of newborn, unspecified

P39.9 Infection specific to the perinatal period, unspecified
